# A Multidisciplinary Approach to End-Stage Limb Salvage in the Highly Comorbid Atraumatic Population: An Observational Study

**DOI:** 10.3390/jcm13082406

**Published:** 2024-04-20

**Authors:** Karen R. Li, Christian X. Lava, Monique B. Neughebauer, Rachel N. Rohrich, Jayson Atves, John Steinberg, Cameron M. Akbari, Richard C. Youn, Christopher E. Attinger, Karen K. Evans

**Affiliations:** 1 Georgetown University School of Medicine, Washington, DC 20007, USA; 2 George Washington University, Washington, DC 20052, USA; 3 Department of Podiatry, MedStar Georgetown University Hospital, Washington, DC 20007, USA; 4 Department of Vascular Surgery, MedStar Georgetown University Hospital, Washington, DC 20007, USA; 5 Department of Plastic and Reconstructive Surgery, MedStar Georgetown University Hospital, Washington, DC 20007, USA

**Keywords:** lower extremity reconstruction, free tissue transfer, diabetic limb salvage, atraumatic, chronic wound

## Abstract

**Background:** The use of free tissue transfer (FTT) is efficacious for chronic, non-healing lower extremity (LE) wounds. The four pillars of managing patient comorbidities, infection control, blood flow status, and biomechanical function are critical in achieving successful limb salvage. The authors present their multidisciplinary institutional experience with a review of 300 FTTs performed for the complex LE limb salvage of chronic LE wounds. **Methods:** A single-institution, retrospective review of atraumatic LE FTTs performed by a single surgeon from July 2011 to January 2023 was reviewed. Data on patient demographics, comorbidities, preoperative management, intraoperative details, flap outcomes, postoperative complications, and long-term outcomes were collected. **Results:** A total of 300 patients who underwent LE FTT were included in our retrospective review. Patients were on average 55.9 ± 13.6 years old with a median Charlson Comorbidity Index of 4 (IQR: 3). The majority of patients were male (70.7%). The overall hospital length of stay (LOS) was 27 days (IQR: 16), with a postoperative LOS of 14 days (IQR: 9.5). The most prevalent comorbidities were diabetes (54.7%), followed by peripheral vascular disease (PVD: 35%) and chronic kidney disease (CKD: 15.7%). The average operative LE FTT time was 416 ± 115 min. The majority of flaps were anterolateral thigh (ALT) flaps (52.7%), followed by vastus lateralis (VL) flaps (25.3%). The immediate flap success rate was 96.3%. The postoperative ipsilateral amputation rate was 12.7%. **Conclusions:** Successful limb salvage is possible in a highly comorbid patient population with a high prevalence of diabetes mellitus, peripheral vascular disease, and end-stage renal disease. In order to optimize patients prior to their LE FTT, extensive laboratory, arterial, and venous preoperative testing and diabetes management are needed preoperatively. Postoperative monitoring and long-term follow-up with a multidisciplinary team are also crucial for long-term limb salvage success.

## 1. Introduction

In the United States, an estimated 150,000 individuals undergo lower extremity (LE) amputations annually [1]. Amongst high-risk patients undergoing LE amputations, the 5-year mortality ranges from 39% to 70% [2]. This estimate continues to grow, primarily driven by climbing rates of diabetes mellitus (DM) and peripheral vascular disease (PVD). Diabetic foot ulcers are the primary cause of 60 to 80% of non-traumatic LE amputations [3]. This high incidence aligns with the high-risk profile of diabetic patients and their associated comorbidities. These patients are further predisposed to several independent risk factors for LE amputation, including end-stage renal disease (ESRD) requiring hemodialysis, elevated HbA1c, PVD, and neuropathy [4,5]. Furthermore, the risk of eventual LE amputation is exacerbated in patients with PVD, who lack adequate blood flow imperative to wound healing and infection resolution, often resulting in ischemic complications, delayed wound healing, and potential gangrene [6,7].

**Figure 1:**
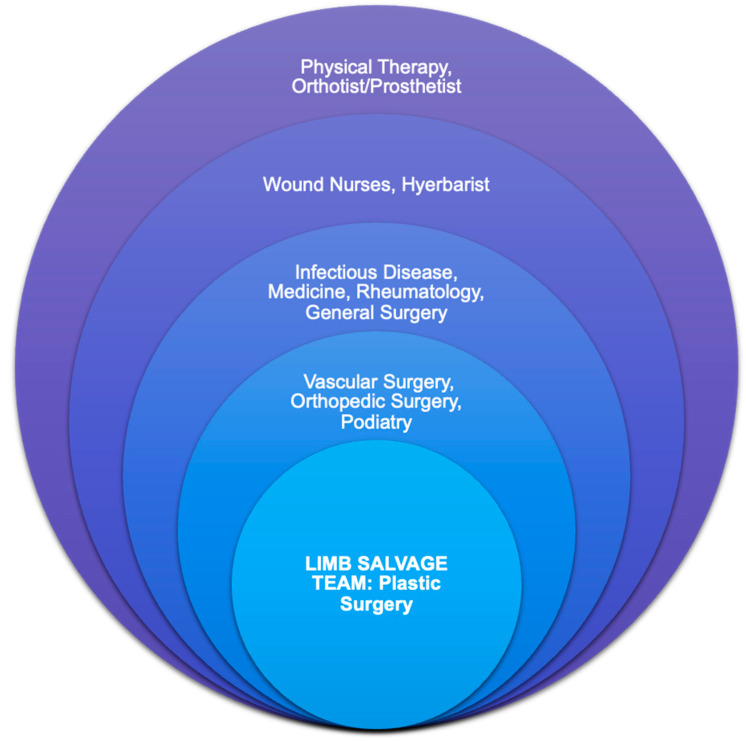
Limb salvage team model in a multidisciplinary wound clinic setting.

Free tissue transfer (FTT) has emerged as an effective surgical intervention for soft tissue reconstruction, with reported success rates of 95 to 99% [8,9,10]. Despite high success rates, the preoperative optimization of comorbidities is crucial to successful outcomes, especially in the non-traumatic LE population. In this setting, the four key pillars of patient factors—infection control, blood flow optimization, and biomechanical function—are critical to consider in the multidisciplinary approach to limb salvage (Figure 1).

The continuous improvements in preoperative planning, intraoperative techniques, and postoperative care have maintained LE FTT as a viable limb salvage option for patients with non-traumatic, highly comorbid, and vasculopathic conditions. We highlight our institution’s multidisciplinary approach and experience with 300 LE FTTs in complex limb salvage.

## 2. Methods

Following institutional review board approval (we conducted a single-institution retrospective review of LE FTTs performed from July 2011 to January 2023), all procedures were performed by the senior author. Demographics, comorbidities, and the Charlson Comorbidity Index (CCI), an index used to quantify the comorbid burden and predict 10-year mortality, were collected [11]. Additional preoperative data included preoperative laboratory values, hypercoagulability testing, the number of debridements prior to FTT, wound characteristics, venous testing results, diagnostic angiogram details, vascular intervention, and home anticoagulant and antiplatelet regimens. Vessel run-off (VRO) was calculated as zero, one, two, or three patent lower extremity vessels from the angiogram. Intraoperatively, data on the type of flap, flap tissue composition, type of arterial anastomosis, use of a saphenous vein interposition graft (sVIG), presence of calcified vessels, and duration of operation were collected. Postoperative complications and long-term outcomes were also collected. Immediate flap success was determined from postoperative day (POD) 0 to 12, and it was defined by flap viability that did not require additional surgical intervention. Immediate postoperative outcomes included rates of takeback from POD zero to seven, partial flap necrosis from POD zero to twelve, hematoma, dehiscence, infection, and donor site complications. Long-term outcomes included rates of postoperative ipsilateral amputation, time to amputation, follow-up duration, ambulatory status, and mortality.

### 2.1. Preoperative Management

**Figure 2:**
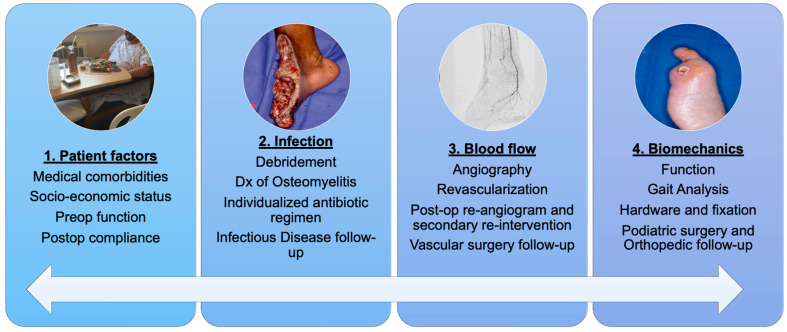
Multidisciplinary limb salvage: 4 critical factors to consider for successful complex soft tissue coverage for the highly comorbid patient.

Our multidisciplinary approach to limb salvage required the careful coordination of plastic, vascular, podiatric, and orthopedic surgeons, hospitalists, and infectious disease specialists (Figure 2).

**Figure 3:**
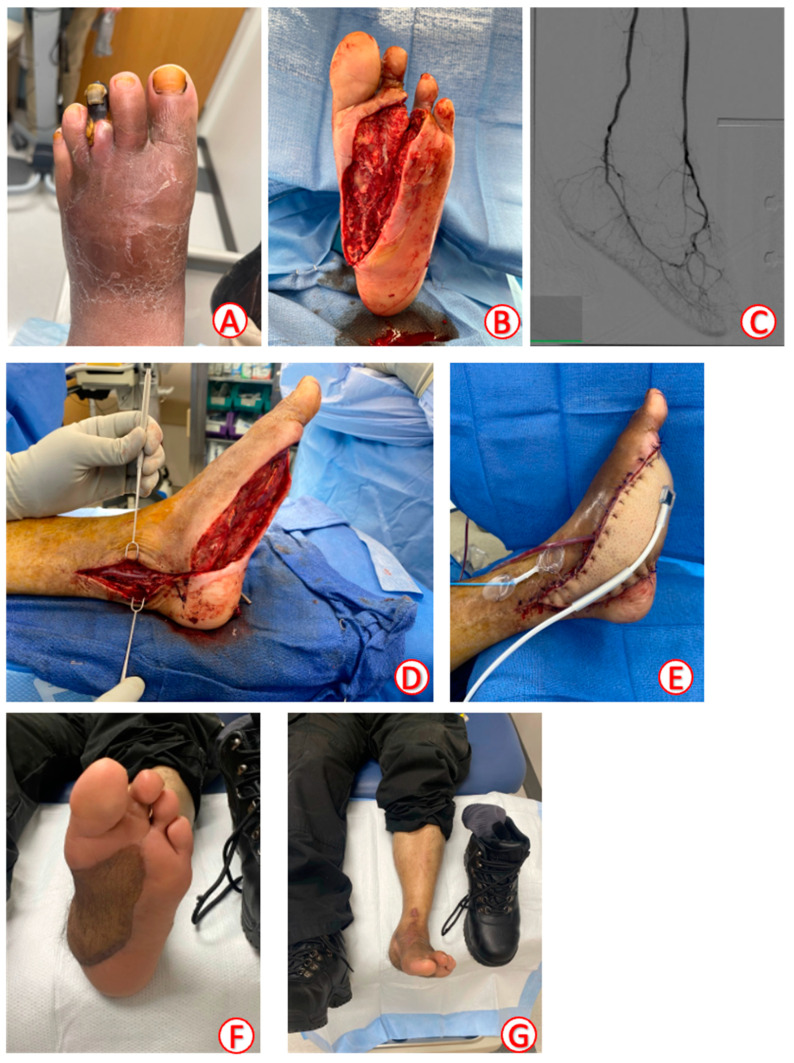
A 53-year-old male with a past medical history of type II diabetes mellitus with peripheral neuropathy A1c 9.3% who presents to the clinic with a left foot infection, abscess, and gas gangrene. (**A**) Patient presents to the clinic with infection of the L foot and gangrene of the 3rd toe. Patient is admitted to the limb surgical service. Patient receives a CT scan, which confirms gas gangrene and shows changes corresponding to osteomyelitis. (**B**) Photograph post incision and drainage of left foot and open partial third ray amputation. Patient presents with a large plantar foot defect measuring approximately 13 × 7 cm in size, extending from second webspace anteriorly and continuing plantarly and medially to the medial aspect of calcaneal region with exposed intrinsic muscles of the foot. Post-debridement cultures are positive for polymicrobial infection. Biopsy of the left toe is taken and reported to show evidence of acute osteomyelitis. Patient receives four additional debridements prior to free flap. (**C**) Photograph of patient’s diagnostic angiogram showing 2-vessel run-off with widely patent anterior tibial (AT) artery, which continue onto the foot as a dorsalis pedis artery (arrow), widely patent posterior tibial (PT) artery which continues onto the foot as plantar vessels, and intact pedal arch (arrow). Return to the OR for second debridement; photograph of post-excisional debridement (obtained from chart). (**D**) Intraoperative photograph of recipient site dissection. Incision placed 2 cm posterior to medial malleolus where strong doppler signal is heard from the posterior tibial artery. Posterior tibial artery measures 2.5 mm in caliber with evidence of moderate calcifications throughout the vessel. (**E**) Intraoperative photograph of free anterolateral (ALT) flap with end-to-side anastomosis to PT. Implantable Cook Doppler and Vioptix placed for postoperative monitoring. (**F**) Follow-up in clinic 9.6 months post-op showing well-healed free flap. (**G**) Follow-up in clinic 9.6 months post-op showing patient is ambulating with regular footwear.

Figure 3 illustrates a patient case with preoperative testing, intraoperative details, and postoperative follow-up.

Preoperatively, patients considered for microvascular reconstruction were selected based on their overall health status, functional prognosis, and ability to tolerate general anesthesia for a prolonged operation. In order to decrease the risk of infection, LE wound beds were debrided serially until post-debridement cultures, taken as swab cultures, were negative or the wound appeared clinically ready for closure. In cases of suspected infection, infected hardware or foreign objects were removed and adjunct culture-directed antibiotic therapy is started based on a dedicated surgical infectious disease team that manages antibiotic therapy. This team determines the length and type of antibiotics based on culture results. Patients with suspected osteomyelitis underwent bone biopsies along with aggressive debridement to healthy, bleeding bone (Figure 3A,B). In-between debridements, wet-to-dry dressing changes or vacuum-assisted closures were used for local wound care. Proceeding with free flap reconstruction depended on the presence of negative cultures, absence of clinical signs of infection, and adequate response to local wound care [12].

All patients were treated in an inpatient hospital setting and underwent routine testing prior to FTT, which included preoperative labs and a hypercoagulability panel [13,14]. Vascular imaging included both routine angiography and venous testing by duplex ultrasound (US) (Figure 3C). Diagnostic endovascular angiography by the vascular surgery team was performed to identify arterial abnormalities and the necessity for therapeutic endovascular intervention. Vascular interventions, which occurred 5 to 10 days before FTT, included balloon angioplasty, vessel stenting, or open vascular bypass [15]. Venous testing was performed to identify signs of reflux or subclinical thromboses [16], which helped guide recipient vessel decision making.

### 2.2. Intraoperative Management

Considerations for flap selection have been described in a previous study [17]. The type of flap on weight-bearing surfaces depended on the depth of the wound and the availability of the donor site (Figure 3D). A longitudinal slit arteriotomy with end-to-side (ETS) anastomosis for the arterial anastomosis followed by dual venous anastomoses to increase flap outflow were performed, when applicable [18]. An sVIG was used in cases when both the flap and recipient arteries were severely calcified [19,20]. The saphenous vein can be easily identified in the same field by undermining anterior-proximal to the medial malleolus. The Cook Doppler (Cook Medical, Bloomington, IN, USA) devices [21,22,23] were placed intraoperatively during the flap inset to monitor pedicle integrity and flap perfusion (Figure 3E).

### 2.3. Postoperative Management

Following LE FTT, the careful monitoring and stepwise progression of function in the postoperative period was observed. Patients were examined clinically and underwent frequent flap perfusion assessments with a handheld doppler device or Vioptix (ViOptix Inc., Newark, CA, USA) probe for the first five PODs. Patients began a progressive dangling protocol beginning POD five to seven. Patients were discharged home once the flap was deemed stable with 15 min of gravitational dependence, typically around day 10 or 11 [24]. At one month postoperatively, patients started a graduated physical rehabilitation program. Weight bearing in a controlled ankle movement walker boot was allowed four to six weeks after surgery, depending on the location of the flap. Patients were monitored closely with frequent returns to follow-up at the wound clinic (Figure 3F,G). Patients were seen by a prosthetist during their follow-ups to be fitted for custom shoes and/or inserts. Additionally, they were assessed for their LE vascular status, evidence of new ulcerations, and ambulatory function and received appropriate ongoing clinical care for comorbidity management, any complications, ambulation status, and functional ability.

### 2.4. Statistical Analysis

Descriptive statistics were calculated for all patient data. Normally distributed continuous variables, described by means and standard deviations, and non-normally distributed continuous variables, described by the median and interquartile range (IQR), were determined by the Shapiro–Wilk normality test. Categorical variables were reported as frequencies and percentages. Statistical analysis was performed using StataBE Software Version 17 (StataCorp, College Station, TX, USA).

## 3. Results

### 3.1. Demographics

A total of 300 patients who underwent LE FTT were included in our analysis. Patient demographics are described in Table 1.

The average age and median body mass index (BMI) were 55.9 ± 13.6 years and 28.5 (IQR: 7.7) kg/m^2^, respectively. The majority were male (n = 212, 70.7%). Overall, 36.3% of patients had a history of smoking. The median CCI was 4 (IQR: 3), which represents a 53% risk of mortality in ten years [25]. The most prevalent comorbidities present were diabetes (54.7%), followed by PVD (35%) and chronic kidney disease (CKD: 15.7%). Overall, 9% of patients had a history of Charcot arthropathy. Preoperative testing results showed that 76.7% of patients were positive for hypercoagulability traits. At home, 6.7% were on a home anticoagulation regimen and 38% were on antiplatelets prior to LE FTT.

### 3.2. Preoperative Details

Preoperative laboratory testing results are shown in Table 2.

Patients had an elevated average erythrocyte sedimentation rate (ESR) of 66.3 ± 39.7 mm/h, elevated average c-reactive protein (CRP) of 16.5 ± 35.5, elevated average hemoglobin A1c (HbA1c) of 6.4 ± 2.6%, low average albumin of 3.1 ± 1.0, and low average hemoglobin levels (Hgb) of 10.0 ± 1.7.

Wound presentation is shown in Table 3.

The median wound size is 77.5 cm^2^ (IQR = 72). The majority of wounds are located in the ankle area (n = 96, 32%), followed by hindfoot (n = 65, 21.7%) and lower leg (n = 61, 20.3%) defects. From hospital admission to FTT, the median number of serial debridements performed is 3 (IQR = 1), with an average of ten days (IQR = 8.5) between initial debridement and FTT. All patients receive a diagnostic preoperative angiogram per protocol, at a median of 8 days (IQR = 9) prior to FTT (Table 4).

The vascular status of patients in our study showed that 56.5% had 3-VRO, 26.9% had 2-VRO, 13.3% had 1-VRO, and 3.4% had 0-VRO. Endovascular intervention was indicated in 18.7% of the patients prior to FTT and vascular bypass was performed in 1.3%. On preoperative venous testing, 29.8% of patients had evidence of deep venous reflux (VR), 11.1% had superficial VR, and 34.3% had both types of VR. Venous testing also detected abnormalities of venous thromboses (VTs), showing that 5.2% of patients had evidence of deep VTs, 10.0% had superficial VTs, and 1.7% had both deep and superficial VTs.

### 3.3. Operative Details

Operative details are provided in Table 5.

The majority of flaps were anterolateral thigh (ALT) flaps (52.7%), followed by vastus lateralis (VL) flaps (25.3%). Collectively, 54.3% of FTTs utilized fasciocutaneous free flaps, followed by 32.3% muscle-free flaps. For arterial anastomoses, end-to-side (ETS) anastomosis was used in 86.7% of all patients (n = 260). Intraoperative findings of arterial calcification were 28.0% in our study, with 11.7% of patients receiving an sVIG. The rate of intraoperative thrombosis was 1.3% and the rate of intraoperative anastomosis was 7.7%. The majority of patients received two venous anastomoses (81.0%). Postoperatively, 81% of patients received a Cook Doppler (Cook Medical, Bloomington, ID, USA) for monitoring and 31.0% with ViOptix (ViOptix, Newark, CA, USA) monitoring.

### 3.4. Postoperative Complications and Long-Term Outcomes

Postoperative outcomes are listed in Table 6.

The immediate flap success rate was 96.3%, with a takeback rate of 6.0%. Time to takeback was 1.5 days (IQR: 7). Overall, 3.3% of patients experienced partial flap necrosis from POD 0 to 12. By a mean follow-up duration of 15 (IQR: 24.3) months, 5.3% of patients had hematomas, 15.7% had dehiscence, 14.0% had infection, and 7.7% had a donor site complication. Overall, 84.3% of patients reached ambulation status either independently or with an assistive device. The postoperative ipsilateral amputation rate in this population was 12.7% at a median follow-up time of 15 months (IQR: 24.3 months).

## 4. Discussion

In the management of chronic, non-traumatic wounds requiring LE FTT, a multidisciplinary vasculo-plastic approach is essential for achieving optimal outcomes in limb salvage efforts, particularly in the highly comorbid patient population. This necessitates a comprehensive strategy that includes preoperative optimization, intraoperative surgical techniques, and rigorous postoperative care, complemented by long-term follow-up in a multidisciplinary wound clinic. In our institution’s coordinated vasculo-plastic approach to limb salvage, we characterize the essential elements of LE reconstruction for this patient group. We highlight the medical management and key guidelines that have driven our high success rates in LE FTT. Additionally, we explore future directions in the field of limb salvage.

### 4.1. Managing Patient Comorbidities

It is well established that patients undergoing limb salvage for chronic LE wounds carry a high comorbidity burden [26,27,28,29,30]. Unsurprisingly, 54.7% of our total cohort had a history of diabetes and 35% had a history of PVD. The average HbA1c among patients was 6.4, falling between the American Diabetes Association’s cut-off for pre-diabetes and diabetes [31]. Diabetic foot ulcers (DFUs) are a leading cause of hospitalization [32], and the additional diagnosis of PVD has been identified as an independent predictor of limb loss in this population [12,33,34,35]. The combined impact of diabetes and PVD accelerates the pathogenesis of atherosclerosis and leads to critical limb ischemia, emphasizing the importance of diabetes management. Preoperative and postoperative hyperglycemia, defined as blood glucose greater than 200 mg/dL, and elevated Hb1Ac levels above 6.5% have been found to be independent risk factors associated with increased rates of dehiscence [36].

Our study reveals a high prevalence of vasculopathy among patients, with 8.3% with a history of venous thromboembolism (VTE), 76.7% testing positive for hypercoagulability traits, and 38% on a home antiplatelet regimen. Some independent risk factors to VTE include age, BMI, major surgery, and a history of prior superficial vein thrombosis. More importantly, hospitalized patients have a >100-fold increased incidence of VTE [37,38,39,40]. To mitigate the risks of thrombosis in patients with such a high prevalence of vascular disease, we routinely conduct a preoperative hypercoagulability panel and implement a risk-stratified anticoagulation protocol. These measures have previously been shown to increase the success of LE FTT at our institution and lower rates of flap loss among risk-stratified patients [14,41,42]. By risk-stratifying patients into low-, moderate-, and high-risk categories and treating them with appropriate AC regimens, we can balance antithrombotic benefits with the risk of bleeding [41]. Patients that have suboptimal Hgb levels at baseline are at further risk of bleeding intraoperatively [43]. Despite the significantly higher thrombogenic risk in these patients, careful attention to anticoagulation protocols can support low rates of intraoperative thrombosis and decrease the incidence of postoperative thrombotic complications. Taken together, our results highlight the importance of the preoperative identification of patients at high thrombogenic risk.

### 4.2. Infection Control

Infection is a leading cause of flap failure, contributing to a non-healing status that often progresses to amputation in chronically infected patients [44,45,46]. The rate of post-FTT infection in our study was 14.0%. The majority of patients in our study present with multiple comorbidities, including a history of diabetes and immunosuppression, that impede effective wound healing and place them at high risk for infectious complications [47,48,49]. Biofilm formation contributes to infection and has been found in 60% to 78.2% of chronic wounds [50]. The biofilm’s pathogenicity intensifies as it diversifies and becomes physically attached to the wound, enabling the infection to evade traditional antimicrobial therapies [51]. Because the biofilm exists both on the surface of the wound and in deeper tissues, surgical debridement is necessary in these patients to remove the devitalized tissue and bacteria that impede healing and promote infection [52]. However, single debridements prior to FTT do not always achieve complete sterility of the wounds [53]. Previously, it has been shown that positive post-debridement cultures (PDCs) prior to local muscle flap coverage independently predicted wound non-closure at 90 days [54]. In the present study, we observed that a median of three serial debridements performed over a median of 10 days before FTT were required to reach adequate sterility of the wound. Limb salvage protocols at our institution have evolved to ensure patients are serially debrided until PDCs are negative to decrease infection risk postoperatively.

### 4.3. Optimizing for Diseased Vasculature

In addition to the challenges posed by thrombogenic risk factors, the sequelae of PVD further complicate LE FTT in this population. This pathology presents with multi-level occlusions in arterial vessels and damages venous valves, leading to chronic venous insufficiency, venous reflux, and DVT [55,56,57]. It is crucial that a multidisciplinary vascular and plastic surgery approach is utilized in this setting to understand the patient’s preoperative vascular status. A key part of this understanding is our institution’s protocol for a preoperative diagnostic angiogram in LE FTT patients. The utility of this imaging is to determine vessel patency, indicate the need for further endovascular intervention, and guide optimal recipient artery selection [16]. Revascularization has been shown to improve wound healing and limb salvage rates in patients at risk for critical limb ischemia [58,59,60,61,62]. Endovascular intervention by percutaneous balloon angioplasty is preferred due to its ability to re-intervene if the vessel re-stenoses [63,64]. In our study, prior to endovascular intervention, 56.5% of patients had 3-VRO, 26.9% of patients had 2-VRO, 13.3% of patients had 1-VRO, and 3.4% of patients had 0-VRO. Post-endovascular intervention, patients were revascularized and showed overall improvements in VRO, with a greater proportion of patients with 3-VRO (62.9%) and 2-VRO (27.2%), and a lower proportion of patients with 1-VRO (9.2%) and 0-VRO (0.7%).

Preoperative venous testing also helps in detecting venous abnormalities that may put the patient at higher risk of complications. Rates of thrombosis have been found to range from 5 to 30% in free tissue transfer [65], with other studies citing an incidence of 61.7% [66]. Venous testing allows for the detection of venous thromboses in the deep and superficial venous systems, which we found to occur at a rate of 16.9% in our study. This testing is particularly important in patients who have a baseline status of vasculopathy, as it gives the surgical team a better understanding of the vessels that are diseased and informs the use of a healthy venous system for intraoperative anastomosis. In order to maximize outflow and decrease risks of venous congestion, a two-vein anastomosis is preferred [67,68,69]. Current studies support the use of a second vein anastomosis in microsurgical procedures if technically feasible [70,71,72,73].

In addition to the efforts required to optimize a patient’s vascular status preoperatively, patients are still at high risk for ischemic complications given their baseline disease and challenged perfusion status. The rates of takeback from POD zero to seven were 6.0%, of which 3.7% were salvaged. Postoperative flap monitoring is crucial for flap survival, especially during first 72 h following surgery [74,75]. At our institution, we use an implantable Cook–Swartz Venous Doppler (Cook Medical, Bloomington, ID, USA) device, a handheld bedside doppler, ViOptix (ViOptix, Newark, CA, USA), or a combination of the three to monitor hemodynamics of the LE FTT. Previous studies have demonstrated that the implantable Cook Doppler significantly decreases rates of takeback and improves rates of flap salvage [76,77]. This monitoring is especially important for muscle flaps, where vascular compromise may not be immediately apparent due to the muscle’s ability to retain blood volume without signs of congestion. Likewise, the use of ViOptix (ViOptix, Newark, CA, USA), a near-infrared spectroscopy tissue oximetry technology for postoperative noninvasive flap perfusion monitoring, has been shown to be an effective means to reduce rates of takeback via capturing drops in O_2_ saturation. With these hemodynamic monitors in place, we observed a rate of partial necrosis from POD 0 to 12 of 3.3%, which demonstrates the utility of intensive postoperative monitoring to achieve high flap success rates.

### 4.4. Long-Term Limb Salvage Outcomes

In addition to the preoperative and intraoperative protocols previously described, patients who receive LE FTT at our multidisciplinary wound clinic are monitored closely after surgery. Postoperatively, patients were seen at our clinic at a median of 15 months for follow-up visits. Consistent postoperative surveillance is critical for these patients to ensure healing progress, adherence to offloading protocols, and the proper management of comorbid conditions [31,78]. Re-ulceration or flap failure can occur if underlying biomechanical corrections are not addressed or excessive pressure is placed on the LE FTT prior to full recovery [79,80,81,82,83]. Longitudinal management addressing patient comorbidities, decreasing risks of infection, maintaining patent vascular status, and restoring biomechanical function are critical in preventing progression to amputation. Ultimately, through a comprehensive and multidisciplinary approach to LE FTT for the chronic wound population, we demonstrate that the long-term outcomes of these procedures are beneficial for patients. Over 80% of patients achieved ambulation either independently, or with assistance after their LE FTT. The importance of maintaining consistent long-term follow-up is reflected in the high success rates of ambulation for this population in limb salvage.

### 4.5. Limitations

This study was limited to its retrospective study design. However, in our single-institutional review, we provide a comprehensive overview of a multidisciplinary approach to lower extremity free tissue transfer.

## 5. Conclusions

Successful limb salvage is possible in a highly comorbid patient population with a high prevalence of diabetes mellitus, peripheral vascular disease, and end-stage renal disease. In order to optimize patients prior to their LE FTT, extensive laboratory, arterial, and venous preoperative testing and diabetes management are needed preoperatively. Postoperative monitoring and long-term follow-up with a multidisciplinary team are also crucial for long-term limb salvage success.

## Figures and Tables

**Table 1:** Patient Demographics.

		Total, *n* (%)
Age, mean ± SD		55.88 ± 13.62
Sex		
	Male	212 (70.7%)
	Female	88 (29.3%)
Race		
	White	134 (44.8%)
	Black or African American	131 (43.8%)
	Hispanic	10 (3.3%)
	Asian	6 (2.0%)
	Other/unknown	18 (6.0%)
BMI (kg/m^2^), median (IQR)		28.5 (7.7)
Hospital LOS, median (IQR)		27 (16)
Postop LOS, median (IQR)		14 (8.5)
Smoking		
	Never smoker	191 (63.7%)
	Former	69 (23%)
	Current	40 (13.3%)
CCI, median (IQR)		4 (3)
DM		164 (54.7%)
PVD		105 (35%)
ESRD		15 (5%)
VTE		25 (8.3%)
Transplant history		9 (3%)
MI		12 (4%)
CVA/TIA		14 (4.67%)
Malignancy history		34 (11.33%)
CKD		47 (15.67%)
CHF		16 (5.33%)
COPD		9 (3%)
Hypercoagulability		230 (76.7%)
Home AC		20 (6.67%)
Home AP		114 (38%)

Abbreviations: IQR: interquartile range; SD: standard deviation; CCI: Charlson Comorbidity Index; DM: diabetes; PVD: peripheral vascular disease; CKD: chronic kidney disease; ESRD: end-stage renal disease; VTE: venous thromboembolism; MI: myocardial infarction; CVA/TIA: cerebrovascular disease/transient ischemic attack; COPD: chronic obstructive pulmonary disease; CHF: chronic heart failure; AC: anticoagulation; AP: antiplatelet.

**Table 2:** Preoperative Labs.

	Total
WBC (×10^9^/L), mean ± SD	7.95 ± 2.71
ESR (mm/h), mean ± SD	66.28 ± 39.69
CRP (mg/dL), median (IQR)	16.5 (35.5)
HgbA1c (%), median (IQR)	6.4 (2.6)
Albumin (g/dL), median (IQR)	3.1 (1.0)
Prealbumin (mg/dL), mean ± SD	19.0 ± 7.0
Hgb on DOS (g/dL), mean ± SD	9.98 ± 1.71
Platelet count (/microL), median (IQR)	274 (131)

Laboratory values at our institution are automatically marked out of range if they are not within the following ranges: INR (0.8–1.2), Hgb (12.5–16.5 gm/dL), WBC (4.0–10.8 × 10^9^/L), platelet count (145–400/µL), CRP (0–3), prealbumin 20–40 mg/dL, albumin (3.5–5 g/dL), HgbA1c (4.2–5.6%). High and low glucose represent the highest and lowest measured glucose during the postoperative stay.

**Table 3:** Wound presentation and details.

		Total, *n* (%)
Wound area (cm^2^), median (IQR)		77.5 (72)
Wound location		
	Forefoot	55 (18.3%)
	Midfoot	50 (16.7%)
	Hindfoot	65 (21.7%)
	Ankle	96 (32%)
	Lower leg	61 (20.3%)
	Knee	12 (4%)
	TMA site	36 (12%)
	BKA stump	4 (1.33%)
	Anterior leg	54 (18%)
	Posterior leg	43 (14.3%)
	Plantar foot	58 (19.3%)
	Dorsal foot	47 (15.7%)
	Medial leg	55 (18.3%)
	Lateral leg	59 (19.7%)
Charcot arthropathy		27 (9%)
Total debridements, median (IQR)		3 (1)
Time from initial DBT to FTT (days), median (IQR)		10 (8.5)

Abbreviations: TMA: transmetatarsal amputation; BKA: below-knee-amputation; DBT: debridement; FTT: free tissue transfer.

**Table 4:** Preoperative Vascular Imaging.

		Total, *n* (%)
Preoperative LE angiogram *		294 (98.0%)
Time from angiogram to FTT (days), median (IQR)		8 (9)
Anterior tibial artery		
	Patent	214 (73.0%)
	Occluded	52 (17.8%)
	Reconstituted	27 (9.2%)
Posterior tibial artery		
	Patent	222 (75.77%)
	Occluded	55 (18.77%)
	Reconstituted	16 (5.46%)
Peroneal artery		
	Patent	255 (87.03%)
	Occluded	26 (8.87%)
	Reconstituted	12 (4.1%)
Dorsalis pedis		
	Patent	236 (89.06%)
	Occluded	18 (6.79%)
	Reconstituted	11 (4.15%)
Vessel run-off, initial		
	3	166 (56.46%)
	2	79 (26.87%)
	1	39 (13.27%)
	0	10 (3.40%)
Endovascular interventions		56 (18.67%)
	Balloon angioplasty	55 (98.21%)
	Stent placement	3 (5.36%)
Vascular bypass		4 (1.33%)
Time from bypass to FTT (days), median (IQR)		16 (1.5%)
Time from intervention to FTT (days), median (IQR)		9.5 (8)
Venous Mapping **		233 (77.7%)
Venous reflux		
	None	45 (24.9%)
	Deep	54 (29.8%)
	Superficial	20 (11.1%)
	Both	62 (34.3%)
Venous thrombosis		
	None	192 (83.1%)
	Deep	12 (5.2%)
	Superficial	23 (10.0%)
	Both	4 (1.7%)

* 6 patients did not receive preoperative angiograms; ** 67 patients did not receive venous testing, due to hospital delays and different orders.

**Table 5:** Intraoperative Flap Details.

		Total	
Flap type			
	ALT/AMT	158 (52.7%)	
	Vastus lateralis	76 (25.3%)	
	Gracilis	3 (1.0%)	
	Rectus Femoris	3 (1.0%)	
	Radial Forearm	6 (2.0%)	
	Latissimus Dorsi	14 (4.7%)	
	Parascapular	1 (0.3%)	
	MSAP	4 (1.3%)	
	MFC	1 (0.3%)	
	Rectus Abdominis	4 (1.3%)	
	Free Fibular	1 (0.3%)	
	SCIP	1 (0.3%)	
Flap tissue composition			
	Chimeric	28 (9.3%)	
	Adipofascial	7 (2.3%)	
	Fasciocutaneous	163 (54.3%)	
	Muscle	97 (32.3%)	
	Myocutaneous	6 (2.0%)	

Abbreviations: ALT: anterolateral thigh; AMT: anteromedial thigh, MSAP: medial sural artery perforator; MFC: medial femoral condyle; SCIP: superficial circumflex iliac artery perforator.

**Table 6:** Postoperative Outcomes.

	Total, *n* (%)
Immediate flap success	289 (96.3%)
Takeback (POD0–7)	18.0 (6.0%)
Time to takeback (days), median (IQR)	1.5 (7)
Flap salvage	11 (3.7%)
Partial flap necrosis (POD0–12)	10 (3.3%)
Hematoma	16 (5.3%)
Dehiscence	47 (15.7%)
Infection	42 (14.0%)
Donor site complication	23 (7.7%)
Postoperative ipsilateral amputation	38 (12.7%)
Time to amputation (days), median (IQR)	169 (220)
Postoperative contralateral amputation	4 (1.3%)
Follow-up duration (months), median (IQR)	14.95 (24.3)
Time to ambulation (months), median (IQR)	3.2 (6.0)
Ambulatory	253 (84.3%)
Mortality	22 (7.4%)

Abbreviations. POD: postoperative day.

## Data Availability

The datasets presented in this article are not readily available, because of HIPAA concerns.
